# Protoporphyrin IX distribution following local application of 5-aminolevulinic acid and its esterified derivatives in the tissue layers of the normal rat colon

**DOI:** 10.1054/bjoc.2001.2124

**Published:** 2001-11

**Authors:** E Endlicher, P Rümmele, F Hausmann, R Krieg, R Knüchel, H C Rath, J Schölmerich, H Messmann

**Affiliations:** Department of Internal Medicine I, Institute of Pathology, Institute of Inorganic Chemistry, University of Regensburg, Regensburg, 93042, Germany

**Keywords:** 5-aminolevulinic acid, 5-aminolevulinic esters, protoporphyrin IX, fluorescence, colon

## Abstract

Photodynamic diagnosis and especially therapy after sensitization with 5-aminolevulinic acid (ALA) is hampered by limitations of uptake and distribution of ALA due to its hydrophilic nature. Chemical modification of ALA into its more lipophilic esters seems to be promising to overcome these problems. The aim of the present study was to investigate the comparative kinetics of protoporphyrin IX (PPIX) fluorescence in rat colonic tissue after topical application of ALA and its esterified derivatives, ALA-hexylester (h-ALA), ALA-methylester (m-ALA) and ALA-benzylester (b-ALA). Fluorescence intensity induced by PPIX in normal colonic tissue was quantified using fluorescence microscopy at 1, 2, 4, 6 and 8 h after sensitization. Mucosa exhibited higher fluorescence levels compared to the underlying submucosa or smooth muscle. Peak fluorescence intensities were seen 4 h after local sensitization with 86.0 mol ml^−1^ ALA (513 ± 0.57 counts per pixel), 6.6 mol ml^−1^ m-ALA (508 ± 35.50) and 4.8 mol ml^−1^ h-ALA (532 ± 128.80), and 6 h after sensitization with 4.6 mol ml^−1^ b-ALA (468 ± 190.27). A 13–18 times lower concentration of ALA esters was required for fluorescence intensities reached with ALA alone. A similar degree of the fluorescence ratio between mucosa and muscularis (5–6:1) was detected for ALA and its derivatives. The time point of the maximum value of this ratio was consistent with peak fluorescence levels for ALA and each ALA-ester. The clinical feasibility and the advantages of topical ALA ester-based fluorescence for detection of malignant and premalignant lesions need further investigations. © 2001 Cancer Research Campaign   http://www.bjcancer.com


British Journal of Cancer (2001) 85(10), 1572–1576
© 2001 Cancer Research Campaign
doi: 10.1054/ bjoc.2001.2124, available online at http://www.idealibrary.com on IDEAL

### Protoporphyrin IX distribution following local application of 5-aminolevulinic acid and its esterified derivatives in the tissue layers of the normal rat colon

**E Endlicher[1], P Rümmele[2], F Hausmann[3], R Krieg[1], R Knüchel[2], HC Rath[1], J Schölmerich[1] and H Messmann[1]**

[1]Department of Internal Medicine I, [2]Institute of Pathology, [3]Institute of Inorganic Chemistry, University of Regensburg, 93042 Regensburg, Germany

**Summary** Photodynamic diagnosis and especially therapy after sensitization with 5-aminolevulinic acid (ALA) is hampered by limitations of uptake and distribution of ALA due to its hydrophilic nature. Chemical modification of ALA into its more lipophilic esters seems to be promising to overcome these problems. The aim of the present study was to investigate the comparative kinetics of protoporphyrin IX (PPIX) fluorescence in rat colonic tissue after topical application of ALA and its esterified derivatives, ALA-hexylester (h-ALA), ALA-methylester (m-ALA) and ALA-benzylester (b-ALA). Fluorescence intensity induced by PPIX in normal colonic tissue was quantified using fluorescence microscopy at 1, 2, 4, 6 and 8 h after sensitization. Mucosa exhibited higher fluorescence levels compared to the underlying submucosa or smooth muscle. Peak fluorescence intensities were seen 4 h after local sensitization with 86.0 mol $ml^{-1}$ ALA (513 ± 0.57 counts per pixel), 6.6 mol $ml^{-1}$ m-ALA (508 ± 35.50) and 4.8 mol $ml^{-1}$ h-ALA (532 ± 128.80), and 6 h after sensitization with 4.6 mol $ml^{-1}$ b-ALA (468 ± 190.27). A 13–18 times lower concentration of ALA esters was required for fluorescence intensities reached with ALA alone. A similar degree of the fluorescence ratio between mucosa and muscularis (5–6:1) was detected for ALA and its derivatives. The time point of the maximum value of this ratio was consistent with peak fluorescence levels for ALA and each ALA-ester. The clinical feasibility and the advantages of topical ALA ester-based fluorescence for detection of malignant and premalignant lesions need further investigations. © 2001 Cancer Research Campaign http://www.bjcancer.com

**Keywords**: 5-aminolevulinic acid; 5-aminolevulinic esters; protoporphyrin IX; fluorescence; colon

Exogenous 5-aminolevulinic acid (ALA) induced protoporphyrin IX (PPIX) fluorescence endoscopy and photodynamic therapy (PDT) are new methods for detection and treatment of premalignant and malignant lesions in the gastrointestinal tract (Gossner et al, 1998; Endlicher et al, 1999, 2001; Overholt et al, 1999). ALA is a prodrug in haem biosynthesis, which is converted into the photosensitizing agent protoporhyrin IX (PPIX). Due to a low ferrochelatase activity in tumour cells, PPIX accumulates selectively in malignant tissue (Peng et al, 1997; Krieg et al, 2000). Furthermore PPIX is associated with only limited skin photosensitivity of 1–2 days (Regula et al, 1995).

In urology fluorescence cystoscopy after local instillation of ALA into the bladder has significantly enhanced the diagnosis of malignant and premalignant bladder lesions (Kriegmair et al, 1996; Filbeck et al, 1999; Koenig et al, 1999). The local application of ALA seems to be a promising alternative approach as well compared to the systemic administration in the detection of malignant and premalignant lesions in the gastrointestinal tract (Endlicher et al, 1999).

However, when using ALA topically for the PDT of malignant and premalignant lesions, this modality appears to be limited by the amount of uptake and penetration of ALA due to its hydrophilic nature. Therefore, some lipophilic molecules such as esterified ALA derivatives have been used both in vitro (Gaullier et al, 1997; Kloek et al, 1998; Casas et al, 1999; Eleouet et al, 2000), and in vivo (Lange et al, 1999), and better effects were achieved, compared to the application of unmodified ALA. The biosynthesis of PPIX induced by ALA esters depends on the aliphatic length of the alcohol used for esterification. While the long-chained ALA-esters (C5–C8) induced a higher PPIX accumulation in human tumour cell lines, the short-chained ALA-esters (C1–C3) were less effective (Gaullier et al, 1997; Washbrook and Riley, 1997), indicating that the mechanism and rate of uptake of ALA and its esterified derivatives may be different. Berg et al demonstrated, that the transport of ALA into human adenocarcinoma cells is mediated by an active transport mechanism via β-amino acid and gamma aminobutyric acid carriers, whereas ALA esters are taken up by transporters other than those used for uptake of ALA or they may penetrate the plasma membrane by passive diffusion (Rud et al, 2000).

The aim of the present study was to investigate the comparative kinetics of PPIX fluorescence in rat colonic tissue for topical application of ALA and its esterified derivatives. Following our preclinical studies on different carcinoma cell lines (CaCO2, SW480 and HT29) with different ALA derivatives we choose h-ALA and b-ALA because the results indicated that the maximum benefit for the formation of PPIX is obtained with these esters (Hausmann et al, 2001). ALA methylester was used for the comparative study since to our knowledge there are no clinical data for short-chained ALA esters available.

*Received 20 February 2001*
*Revised 25 June 2001*
*Accepted 19 July 2001*

*Correspondence to:* H Messman, University of Regensburg;
Tel.: +49-941-944-7001; Fax: +49-941-944-7002
Email: helmut.messman@klinik.uni-regensburg.de

### MATERIAL AND METHODS

Female CD rats approximately 200 g in weight were used for this study. ALA was obtained from medac GmbH, Hamburg, Germany. ALA methyl-, hexyl- and benzylester were synthesized at the Institute of Inorganic Chemistry, University of Regensburg. All experiments were carried out under nitrogen atmosphere. ALA esters were prepared by reacting ALA with the corresponding alcohol in the presence of thionyl chloride giving the ALA esters as the hydrochloride salts (Takeya, 1992). An excess of the freshly destilled thionyl chloride was added dropwise. Then ALA was added to the solution and stirred at 60˚C for 5 hours. The excess of alcohol was evaporated in vacuo. After recrystallization from methanol/diethylether at –20˚C pure ALA esters were obtained as white hydrochloride salts. Identification of the synthesized compounds was based on mass spectroscopy, elemental analysis and nuclear magnetic resonance (NMR). The protocol for animal studies was approved by the animal board of Unterfranken, Germany, adhering to standards required to meet UKCCCR guidelines regarding animal ethics. The animals were anaesthetized with Ketanest©/Rompun© and underwent a small median laparotomy with mobilization of the caecum. After ligation of the terminal ileum the caecum was incised, a probe placed into the lumen to wash out faeces by a lavage with about 50 ml of 0.9% NaCl solution until the fluid became clear. Before sensitization with 10 ml of a sterile solution of ALA (86.0 mol $ml^{-1}$: 14.4 mg $ml^{-1}$; pH 6.8–7.4), m-ALA (6.6 mol $ml^{-1}$; 1.2 mg $ml^{-1}$: pH 8.8–7.4), h-ALA (4.8 mol $ml^{-1}$: 1.2 mg $ml^{-1}$; pH 6.8–7.4) or b-ALA (4.6 mol $ml^{-1}$: 1.2 mg $ml^{-1}$; pH 6.8–7.4) the anus was ligated with a running loop suture. 1, 2, 4 and 6 h after administration of ALA, m-ALA, h-ALA, b-ALA and additionally 8 h after application of b-ALA animals were killed. The entire colon was removed and 7–9 tissue samples stored immediately in liquid nitrogen. In addition, tissue from liver and kidney were sampled. The pieces were prepared in a darkened room in order to avoid photobleaching of the tissue. Frozen sections of 20 μm thickness were cut and stored at –80˚C.

In parallel conventional 5 μm cryostat sections were cut and stained with haematoxylin and eosin (H&E) for histological diagnosis. For analysis of PPIX fluorescence a fluorescence microscope (Axiovert S 100, Zeiss, Oberkochen, Germany) was used. Specimen were illuminated by a mercury arc lamp (360 +/–20 nm) and emitted fluorescence (630 +/–30 nm) was recorded by a high-sensitive (4096 levels of gray) and high-resolution (1317 × 1035 pixel, with a pixel size of 6.8 × 6.8 μm) peltier cooled (–15˚C) CCD-camera (Princeton Instruments NJ, USA), using a 10 × /0.30 PLAN-NEOFLUAR lens. Care was taken to ensure the same experimental setup for each acquisition (exposure time) to allow accurate comparability of fluorescence intensities. Specific tissue layers and whole images (liver, kidney) were analysed using this technique to determine the relative intensities of porphyrin fluorescence, after correction for autofluorescence levels of each respective tissue layer as measured on specimens from unsensitized animals (MetaMorph, Universal Imaging Corp, PA, USA). For confirmation of histology the sections of the microscopic areas of fluorescence imaging were compared with the stained sections.

### RESULTS

M-ALA, h-ALA and b-ALA were used in a dose of 6.6 mol $ml^{-1}$ (1.2 mg $ml^{-1}$), 4.8 mol $ml^{-1}$ (1.2 mg $ml^{-1}$) and 4.6 mol $ml^{-1}$ (1.2 mg $ml^{-1}$). 4 h after administration of the 3 ALA-esters nearly the same intensity of porphyrin fluorescence was achieved using a 13- to 18-fold higher concentration of ALA. Consequently a dose of 86.0 mol $ml^{-1}$ (14.4 mg $ml^{-1}$) ALA was used for the fluorescence kinetic study.

Fluorescence intensity was highest in the mucosa. Figure 1(A–D) shows the fluorescence levels in the mucosa, muscularis mucosa, submucosa and muscularis propria. Following administration of m-ALA and h-ALA fluorescence intensity peaked 4 h after administration. Fluorescence with b-ALA peaked at 6 h and reached a

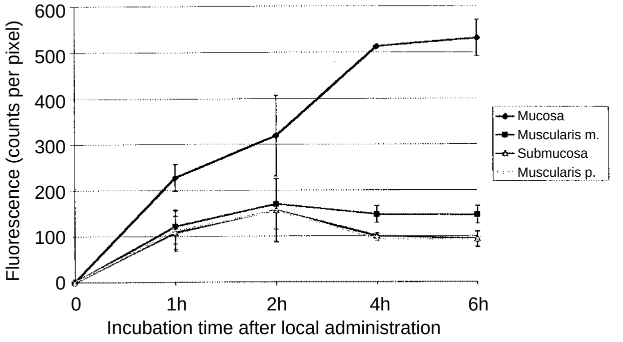

**Figure 1A** Mean fluorescence intensities (± standard deviation) in the mucosa, muscularis mucosae, submucosa and muscularis propria at various times of incubation with ALA (86.0 mol $ml^{-1}$: 14.4 mg $ml^{-1}$)

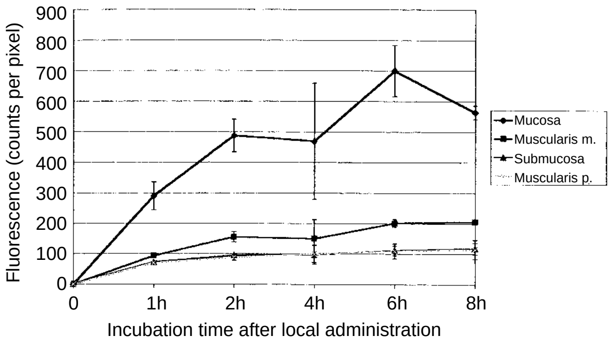

**Figure 1B** Mean fluorescence intensities (± standard deviation) in the mucosa, muscularis mucosae, submucosa and muscularis propria at various times of incubation with b-ALA (4.6 mol $ml^{-1}$: 1.2 mg $ml^{-1}$)

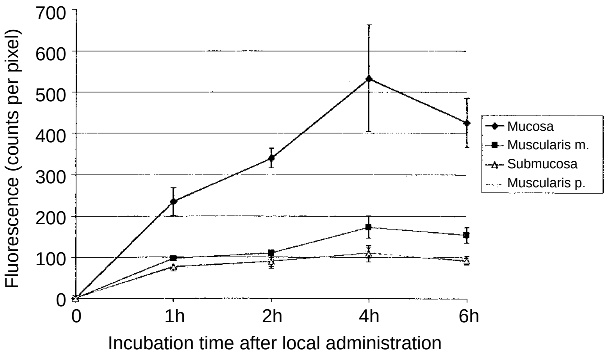

**Figure 1C** Mean fluorescence intensities (± standard deviation) in the mucosa, muscularis mucosae, submucosa and muscularis propria at various times of incubation with h-ALA (4.8 mol $ml^{-1}$: 1.2 mg $ml^{-1}$)

*© 2001 Cancer Research Campaign*

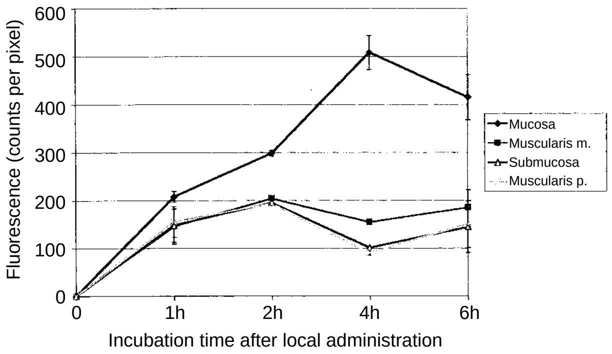

**Figure 1D** Mean fluorescence intensities (± standard deviation) in the mucosa, muscularis mucosae, submucosa and muscularis propria at various times of incubation with m-ALA (6.6 mol ml$^{-1}$: 1.2 mg ml$^{-1}$)

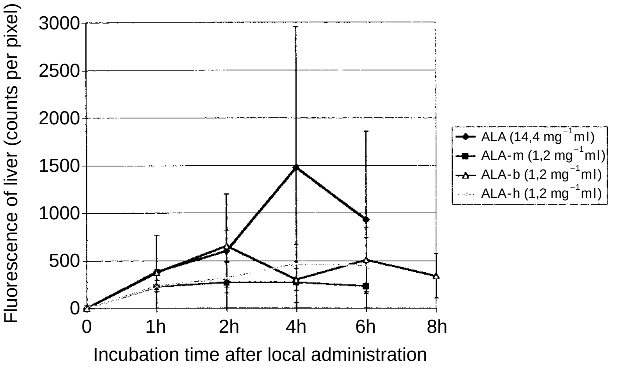

**Figure 3** Mean fluorescence intensities (± standard deviation) in the liver at various times of incubation with different sensitizers

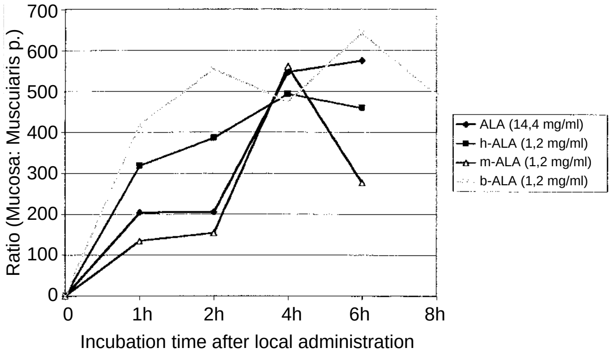

**Figure 2** Ratio of mean fluorescence intensities in mucosa and muscularis propria of rat colon at various times of incubation with ALA and ALA-esters

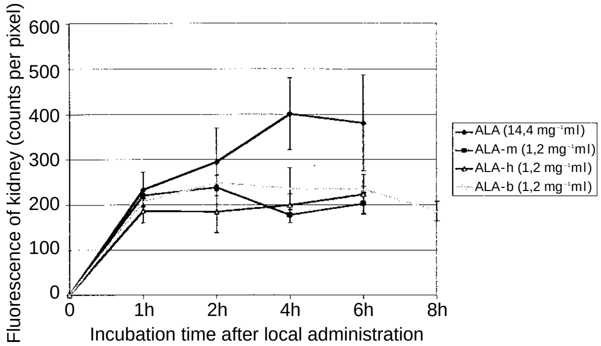

**Figure 4** Mean fluorescence intensities (± standard deviation) in the kidney at various times of incubation with different sensitizers plateau after 4 h for ALA, respectively, but no further time points were examined for sensitization with ALA. Peak fluorescence induced by 4.6 mol ml$^{-1}$ b-ALA (6 h after application) were higher than those 4 h after administration of h-ALA (4.8 mol ml$^{-1}$), m-ALA (6.6 mol ml$^{-1}$) and ALA (86.0 mol ml$^{-1}$).

Different fluorescence intensities of mucosa and muscularis propria as represented by fluorescence ratios were achieved (Figure 2). Following administration of ALA, m-ALA and h-ALA the highest fluorescence ratios between mucosa and muscularis propria occured 4 h after sensitization, following application of b-ALA the maximum fluorescence ratio was seen at 6 h respectively, in accordance to peak mucosal fluorescence levels.

In order to evaluate systemic bioavailability after local application of ALA, h-ALA, m-ALA and b-ALA PPIX fluorescence intensity was also measured in liver and kidney at various time points.

While the esterified derivatives of ALA (h-ALA 4.8 mol ml$^{-1}$, b-ALA 4.6 mol ml$^{-1}$ and m-ALA 6.6 mol ml$^{-1}$) induced no significant increase of PPIX fluorescence intensity in liver and kidney, application of ALA (86.0 mol ml$^{-1}$) resulted in higher values in these organs with a peak or plateau after 4 h (Figures 3 and 4).

Figure 5 shows a typical microscopic fluorescence image of a frozen section of the rat colon after sensitization with ALA and h-ALA.

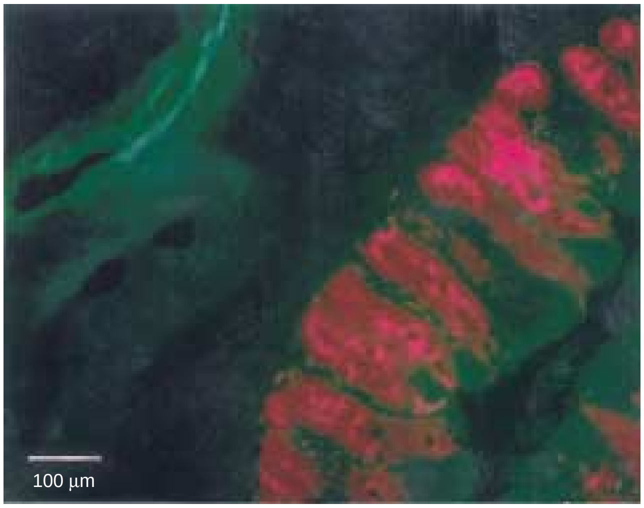

**Figure 5A** Fluorescence image of a frozen section of rat colon 4 h after local administration of ALA (86.0 mol ml$^{-1}$: 14.4 mg ml$^{-1}$): highest fluorescence intensities are found in the mucosa

### DISCUSSION

We have investigated the microscopic distribution of ALA-induced PPIX fluorescence intensity for the esterified derivatives of ALA in the rat colon as well as in liver and kidney. We quantified the PPIX fluorescence at various time intervals after administration of

 *© 2001 Cancer Research Campaign*

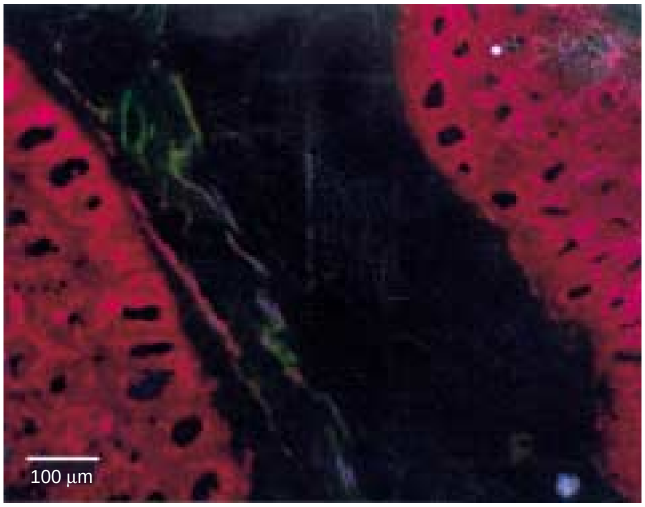

**Figure 5B** Fluorescence image of a frozen section of rat colon 4 h after local administration of ALA-hexylester (4.8 mol ml$^{-1}$: 1.2 mg ml$^{-1}$): Highest fluorescence intensities are found in the mucosa

ALA, m-ALA, b-ALA and h-ALA. Using quantitative fluorescence microscopy the ALA esters induced the same intensity of PPIX fluorescence using a 13- to 18-fold lower concentration as compared to ALA. Consequently, at the time of peak mucosal porphyrin fluorescence intensity we found approximately 3- to 5-fold higher values in the liver after application of ALA compared to its derivatives, indicating a lower systemic bioavailability of the ALA esters. Therefore, with respect to possible side effects the esterified derivatives may be superior compared to ALA.

In accordance to data of intravenous or oral administration of ALA (Loh et al, 1993), following local administration of ALA and its esterified derivatives as presented here, a rapid build up of PPIX fluorescence occurred over the first few hours with a plateau after 4 h. Using a 13- to 18-fold lower concentration of the esterified derivatives, compared to ALA, the temporal kinetics do not show any major difference. Thus endogenous esterases do not seem to have any rate-limiting effect in the tissues investigated. It can be derived from the results with esterified derivatives of ALA that a similar incubation time of ALA and ALA esters is advisable for clinical application in the gastrointestinal tract. This is in contrast to our in vitro data (Hausmann et al, 2001), indicating a more rapid increase of ALA ester-induced PPIX fluorescence leading to shorter incubation times compared to ALA-induced PPIX fluorescence. However Lange et al as well demonstrated shorter necessary incubation times after local ALA ester application for detection of invisible lesions in the bladder (Lange et al, 1999).

In contrast to in vitro data indicating short-chained ALA esters (C1–C3) as less effective in inducing PPIX fluorescence (Gaullier et al, 1997; Washbrook and Riley, 1997), the administration of m-ALA was comparable to h-ALA. The reason for this is unknown. Since the mechanisms and rate of uptake for 5-ALA and its esterified derivatives are not completely understood.

The distribution of ALA- and ALA ester-induced PPIX fluorescence intensity was similar showing localization mainly in the mucosa and approximately 5 times less in the muscularis propria. Bedwell et al investigated the ALA-induced porphyrin content at various time intervals, in both normal and tumour tissue in the rat colon, using fluorescence microscopy and spectroscopy (Bedwell et al, 1992). In accordance to our data presented for the local application mode of ALA, the authors demonstrated that after intravenous sensitization with ALA the fluorescence was mainly localized in the normal mucosa, but was 6 times lower in the submucosal tissue and nearly not detectable in the muscle layer.

Maximum fluorescence ratios of mucosa and musclularis propria were accompanied with peak mucosal PPIX fluorescence with no difference for ALA and its esters.

Loh et al demonstrated in rat colon, that after intravenous and oral administration of ALA mucosal PPIX fluorescence intensities rose to a peak after 4 h, whereas ratios of mean fluorescence levels in mucosa and muscularis propria peaked 1 h after administration (Loh et al, 1993). Therefore, our results indicate that the temporal kinetics of local photosensitization with ALA and its esterified derivatives are preferable compared to systemic sensitization with 5-ALA.

Gil et al investigated the possibility of topical 5-ALA administration for photodynamic therapy of gut cancer in a mice model after instillation of a 5-ALA solution at different concentrations (Gil et al, 1999). The results of fluorescence studies were compared with those obtained in a control group treated with 5-ALA given systemically. They found satisfactory epithelial fluorescence levels and good selectivity between gut layers after intracolonic 5-ALA instillation, however, mean fluorescence intensity was higher after systemic drug application. 5-ALA esters as shown here may overcome this limitation of fluorescence intensity compared to 5-ALA.

In conclusion, the fluorescence kinetic study on the rat colonic tissue indicates that the local application mode of ALA compared to systemic administration may be a promising alternative. In view of clinical data in urology using ALA hexylesters for fluorescence detection of premalignant and malignant lesions in the bladder investigations on selectivity of ALA-esters in malignant and premalignant lesions in the colon are necessary and in progress (Lange et al, 1999).

### ACKNOWLEDGEMENTS

We are grateful to Sabine Dietrich for her excellent technical support. This study was supported by the Deutsche M Crohn/Colitis ulcerosa Vereinigung DCCV e.V.

### REFERENCES

Bedwell J, MacRobert A, Phillips D and Bown S (1992) Fluorescence distribution and photodynamic effect of ALA-induced PPIX in the DMH rat colonic tumour model. *Br J Cancer* **65**: 818–824

Casas A, Batlle AdC, Butler A, Robertson D, Brown E, MacRobert A and Riley P (1999) Comparative effect of ALA derivatives on protoporphyrin IX production in human and rat skin organ culture. *Br J Cancer* **80**: 1525–1532

Eleouet S, Rousset N, Carre J, Bourre L, Vonarx V, Lajat Y, Bejersbergen van Henegouwen G and Patrice T (2000) In vitro fluorescence, toxicity and phototoxicity induced by delta-aminolevulinic acid (ALA) or ALA-esters. *Photochem Photobiol* **71**: 447–454

Endlicher E, Knüchel R, Schölmerich J and Messmann H (1999) Photodynamic diagnosis of dysplasia in longstanding ulcerative colitis after sensitization with 5-aminolevulinic acid. *Gastrointest Endosc* **49**: AB117

Endlicher E, Knüchel R, Hauser T, Szeimies R, Schölmerich J and Messmann H (2001) Endoscopic fluorescence detection of low and high grade dysplasia in Barrett's oesophagus using systemic or local 5-aminolevulinic acid sensitization. *Gut* **48**: 314–319

Filbeck T, Roessler W, Knuechel R, Straub M, Kiel H and Wieland W (1999) Clinical results of the transurethral resection and evaluation of superficial bladder carcinomas by means of fluorescence diagnosis after intravesical instillation of 5-aminolevulinic acid. *J Endourol* **13**: 117–121

Gaullier J, Berg K, Peng Q, Anholt H, Selbo P, Ma L-W and Moan J (1997) Use of 5-aminolevulinic acid esters to improve photodynamic therapy on cells in culture. *Cancer Res* **57**: 1481–1486

Gil M, Woszczynski M, Regula J, MacRobert A, Butruk E and Bown S (1999) Topical versus systemic 5-aminolevulinic acid administration for photodynamic therapy of the colon in B10.RBP mice. *J Biomedical Optics* **4**: 286–291

Gossner L, Stolte M, Sroka R, Rick K, May A, Hahn E and Ell C (1998) Photodynamic ablation of high-grade dysplasia and early cancer in Barrett's esophagus by means of 5-aminolevulinic acid. *Gastroenterol* **114**: 448–455

Hausmann F, Endlicher E, Krieg R, Schölmerich J, Knuechel R, Brunner H and Messmann H (2001) The effects of 5-aminolevulinic acid-esters on ALA-induced protoporphyrin production in human adenocarcinoma cell lines. *Photochem Photobiol* (in press)

Kloek J, Akkermans W and Bejersbergen van Henegouwen G (1998) Derivates of 5-aminolevulinc acid for photodynamic therapy: enzymatic conversion into protoporphyrin. *Photochem Photobiol* **67**: 150–154

Koenig F, McGovern F, Iarne R, Enquist H, Schomacker K and Deutsch T (1999) Diagnosis of bladder carcinoma using protoporphyrin IX fluorescence induced by 5-aminolaevulinic acid. *BJU Int* **83**: 129–135

Krieg R, Fickweiler S, Wolfbeis O and Knuechel R (2000) Cell-type specific protoporpyrin IX metabolism in human bladder cancer in vitro. *Photochem Photobiol* **72**: 226–233

Kriegmair M, Baumgartner R, Knüchel R, Stepp H, Hofstädter F and Hofstetter A (1996) Detection of early bladder cancer by 5-aminolevulinic acid induced porphyrin fluorescence. *J Urol* **155**: 105–110

Lange N, Jichlinski P, Zellweger M, Forrer M, Marti A, Guillou L, Kuchera P, Wagnieres G and Van den Bergh H (1999) Photodetection of early human bladder cancer based on the fluorescence of 5-aminolevulinic acid hexylester-induced protoporphyrin IX: a pilot study. *Br J Cancer* **80**: 185–193

Loh C, MacRobert A, Bedwell J, Regula J, Krasner N and Bown S (1993) Oral versus intravenous administration of 5-aminolevulinic acid for photodynamic therapy. *Br J Cancer* **68**: 41–51

Overholt B, Panjehpour M and Haydek J (1999) Photodynamic therapy for Barrett's esophagus: follow-up in 100 patients. *Gastrointest Endosc* **49**: 1–7

Peng Q, Warloe T, Berg K, Moan J, Kongshaug M, Giercksky K and Nesland J (1997) Aminolevulinic acid-based photodynamic therapy. Clinical research and future challenges. *Cancer* **79**: 2282–2308

Regula J, Robert AM, Gorchein A, Buonaccorsi G, Thorpe S, Spencer G, Hatfield A and Bown S (1995) Photosensitisation and photodynamic therapy of oesophageal, duodenal and colorectal tumours using 5-aminolevulinic acid induced protoporphyrin IX-a pilot study. *Gut* **36**: 67–75

Rud E, Gederaas O, Hoegset A and Berg K (2000) 5-aminolevulinic acid, but not 5-aminolevulinic acid ester, is transported into adenocarcinoma cells by system BETA transporters. *Photochem Photobiol* **71**: 640–455

Takeya H (1992) Preparation of 5-aminolevulinic acid alkyl esters as herbicides. *Cem Abstr* **116**: P189633

Washbrook R and Riley P (1997) Comparison of delta-aminolevulinic acid and its methyl ester as an inducer of porphyrin synthesis in cultured cells. *Br J Cancer* **75**: 1417–1420

 *© 2001 Cancer Research Campaign*
